# Improvement in Post-Autologous Stem Cell Transplant Survival of Multiple Myeloma Patients: A Long-Term Institutional Experience

**DOI:** 10.3390/cancers14092277

**Published:** 2022-05-03

**Authors:** Jordan Nunnelee, Francesca Cottini, Qiuhong Zhao, Muhammad Salman Faisal, Patrick Elder, Ashley Rosko, Naresh Bumma, Abdullah Khan, Srinivas Devarakonda, Don M. Benson, Yvonne Efebera, Nidhi Sharma

**Affiliations:** 1 College of Medicine, The Ohio State University, Columbus, OH 43210, USA; jordan.nunnelee@osumc.edu; 2 Division of Hematology, Department of Internal Medicine, The Ohio State University, Columbus, OH 43210, USA; francesca.cottini@osumc.edu (F.C.); qiuhong.zhao@osumc.edu (Q.Z.); patrick.elder@osumc.edu (P.E.); ashley.rosko@osumc.edu (A.R.); naresh.bumma@osumc.edu (N.B.); abdullah.khan@osumc.edu (A.K.); srinivas.devarakonda@osumc.edu (S.D.); don.benson@osumc.edu (D.M.B.); 3 Roswell Park Comprehensive Cancer Center, Buffalo, NY 14203, USA; mfaisal3@buffalo.edu; 4 Bone Marrow Transplantation & Cellular Therapy, OhioHealth, Columbus, OH 43210, USA; yvonne.efebera@ohiohealth.com

**Keywords:** multiple myeloma, novel agents, older, FISH, ASCT

## Abstract

**Simple Summary:**

The outcomes of patients with multiple myeloma have significantly improved over the years, following autologous stem cell transplant and the introduction of novel agents. In this study, we performed a retrospective survival analysis on newly diagnosed MM patients receiving ASCT from 1992–2016 at the Ohio State University. We observed that newly diagnosed MM patients’ survival and responses to standard of care treatment have improved dramatically since 1992, primarily due to the inclusion of novel and maintenance therapies. There was an improvement in patient remission status, PFS, and OS, suggesting that evolving standards of therapy for MM are enhancing patient outcomes. These findings highlight the importance for newer interventions to build on prior successes.

**Abstract:**

**Multiple** myeloma (MM) represents 1.8% of all new cancer cases in the U.S. While not curable, advances in treatment, including autologous stem cell transplant (ASCT) and maintenance therapy, have dramatically improved progression-free survival (PFS) and overall survival (OS). We performed a retrospective survival analysis on newly diagnosed MM (NDMM) patients receiving ASCT from 1992–2016 at the Ohio State University. A total of 1001 consecutive NDMM patients were eligible. Patients were split into five groups based on historic changes in novel agents for the treatment of MM. Across the years (1992–2016), there was a statistically significant improvement in both PFS (*p* < 0.01) and OS (*p* < 0.01). Significant improvements in both PFS and OS were seen in patients ≤65 years (*p* < 0.001 and *p* = 0.002) and >65 years old (*p* < 0.001 and *p* = 0.001), respectively. Improved PFS and OS were seen in both standard-risk (*p* < 0.001 and *p* < 0.001) and high-risk patients (*p* < 0.001 and *p* = 0.019). The post-transplant response showed statistically significant improvement across the years (*p* < 0.01). Survival rates for NDMM patients have significantly improved primarily due to the inclusion of novel therapies and post-ASCT maintenance.

## 1. Introduction

The outcomes of patients with multiple myeloma (MM) have significantly improved over the years, following autologous stem cell transplant (ASCT) and the introduction of novel agents [1,2]. The median survival of patients with myeloma was less than one year in the 1960s before the introduction of melphalan and other alkylators [1]. The introduction of combination therapy including steroids in the 1980s improved the overall survival to three years, which has since increased three-fold [3].

In the 1980s, ASCT was part of a high-dose chemotherapy for multiple myeloma. In a landmark clinical trial (IFM trial) published in 1996, high-dose chemotherapy with ASCT resulted in higher rates of complete remission (22% vs. 5%), a higher overall response (81% vs. 57%), and a higher survival rate at five years (52% vs. 12%; *p* = 0.03) compared to conventional chemotherapy [4]. This trial established high-dose chemotherapy with ASCT using melphalan as a standard of care in MM treatment for transplant-eligible patients [5]. Although the IFM trial included patients younger than 65 years of age, multiple studies have since been published demonstrating the efficacy and safety of transplants in older patient populations [6,7], and age alone is not a cutoff for transplant eligibility in ASCT guidelines [5].

The goal of induction therapy before ASCT is to achieve quick cytoreduction, reverse end organ damage, and improve performance status [8,9]. The induction therapies have evolved over the years [10]. In the 1990s, the induction treatment was based on conventional chemotherapy, with vincristine, adriamycin, and dexamethasone (VAD) being the most common regimen. Thalidomide was introduced in 1999, bortezomib in 2003, and lenalidomide in 2005 [1]. The post-induction, pre-transplant rate of complete response (CR) improved from less than 10% with VAD to 10–20% with thalidomide-based induction, to 30% in the mid-2000s with the combination of an immune modulator (lenalidomide or thalidomide) and proteasome inhibitor (bortezomib) [11,12,13,14,15,16,17,18]. During the same period, comparison of a three-drug regimen (a PI, an IMiD, and steroids) showed increased depth of response and improvement in PFS and OS compared to two-drug regimens [19,20]. In the landmark SWOG 0777 trial, the combination of three-drug VRD versus two-drug RD led to improved PFS (43 months vs. 30 months, HR 0.7, and *p* = 0.018) and overall survival (75 months vs. 64 months, HR 0.71, and *p* = 0.025) [21]. Sixty-nine percent of patients in the entire cohort were transplant eligible. ASCT has held its place in the era of novel agents, based on the IFM-09 trial that used a three-drug—lenalidomide, bortezomib, and dexamethasone (VRD)—based induction followed by stem cell transplant or VRD therapy alone. Both groups received lenalidomide-based maintenance for one year. The VRD-based induction achieved complete responses in 48% of patients, which increased to 59% after ASCT [22]. The median progression-free survival (PFS) was longer in the transplant group (50 months vs. 36 months, HR 0.65, and *p* < 0.001). Overall survival (OS) was similar between the two groups [22]. Similar trends were seen in a meta-analysis performed by Dhakal et al. on the newly diagnosed MM (NDMM) patients who received ASCT versus no transplant between 2000–2017. The combined hazard ratio (HR) for PFS was 0.55 (95% CI 0.41–0.76; *p* < 0.001), while the combined HR for OS was 0.76 (0.42–1.36; *p* = 0.2). The meta-regression showed a longer follow-up was necessary for a demonstration of benefit of OS [23]. In another study by McCarthy et al., the three-year PFS and OS were 66% and 88%, respectively, for the NDMM patients treated between 2005–2009 with induction therapy using novel drugs, followed by ASCT and maintenance lenalidomide [24]. This study was a meta-analysis of three randomized controlled trials, where NDMM patients received lenalidomide maintenance post-transplantation versus a placebo. The median PFS was 52.8 months for the lenalidomide group and 23.5 months for the placebo group. The median OS was not reached in the lenalidomide group versus 86 months for the placebo group. Newer therapies, including monoclonal antibodies, continue to deepen remission [25]. Additionally, the utility of maintenance therapy post-ASCT has improved post-transplant outcomes [24]. We sought to analyze the survival outcomes over the years of NDMM patients treated with ASCT at the Ohio State University Wexner Medical Center from 1992–2016.

## 2. Patients and Methods

The study included a retrospective chart analysis of 1001 consecutive NDMM patients undergoing their first ASCT during the period between 1992 and 2016 at the Ohio State University. All patients had consented to the use of their medical records, and the study was conducted in accordance with the institutional guidelines with approval of the institutional review board.

Patients were split into five groups based on historic changes in novel agents for the treatment of MM: 1992–1998 (VAD-based induction: group 1), 1999–2002 (thalidomide-based induction: group 2), 2003–2008 (bortezomib- or lenalidomide-based two-drug induction: group 3), 2009–2013 (three-drug induction with VRD; newer medications for relapse, such as carfilzomib and pomalidomide; inconsistent maintenance use: group 4), and 2014–2016 (consistent post-transplant maintenance; newer agents used for relapsed MM, including a daratumumab and Elotuzumab combination: group 5). Fluorescent in situ hybridization (FISH) results were obtained at diagnosis. Patients were considered to have high-risk FISH if any of the following were noted: del17, t(4:14), t(14:16), hypodiploid, and/or 1q abnormalities. Patients with any other abnormalities or a normal FISH were considered to have standard risk.

## 3. Statistical Analysis

Patient characteristics were summarized using descriptive statistics, such as medians and ranges, frequencies, and percentages, and compared among five patient groups using the Mann–Whitney U test for continuous variables and chi-squared or Fisher’s exact test for categorical variables. The primary endpoints were PFS and OS. PFS was defined as time to progressive disease or death from any cause from the date of transplantation, whichever occurred earlier, censoring those who had not relapsed or died at the last follow-up. OS was defined as time from transplantation to death from any cause, censoring those who were still alive at the last follow-up. Kaplan–Meier curves were used to estimate the PFS and OS rates, and the log-rank tests were conducted for the comparisons on equality of OS or PFS functions between subgroups of patients. Univariable Cox proportional hazard models were conducted to estimate the associations between transplant years, patient characteristics, and PFS, as well as OS, and a multivariable cox proportional hazard model was built including significant factors from the univariable analysis to estimate the independent effect of transplant years on PFS and OS. A *p*-value of <0.05 was considered statistically significant. Analyses were performed using Stata 16 (College Station, TX, USA), ver 16

## 4. Results

### 4.1. Patient Characteristics

The median age of all patients at ASCT was 58 years (range: 18–81 years), and 58.4% were male. The median patient age at transplant increased significantly, from 54 to 60 years, from 1992 to 2016 (*p* < 0.001). This was due to older patients getting transplanted, as evident by the increase in patients above 65 years of age from zero to 25.1% in group 1 and group 5, respectively. Caucasians represented 84.9% of patients, and 14.1% were of African descent. Most patients (53.6%) had IgG myeloma, and 19.4% had light chain disease. Of the patients with FISH data, 30% of patients had high-risk disease. An amount of 200 mg/m^2^ melphalan was used in 80.2% of patients as a conditioning regimen. The incidence of CR or VGPR prior to ASCT increased from 5.3% in group 1 to 54.0% in group 5. Other baseline clinical characteristics are provided in Table 1.

### 4.2. Survival Outcomes

**Figure 1:**
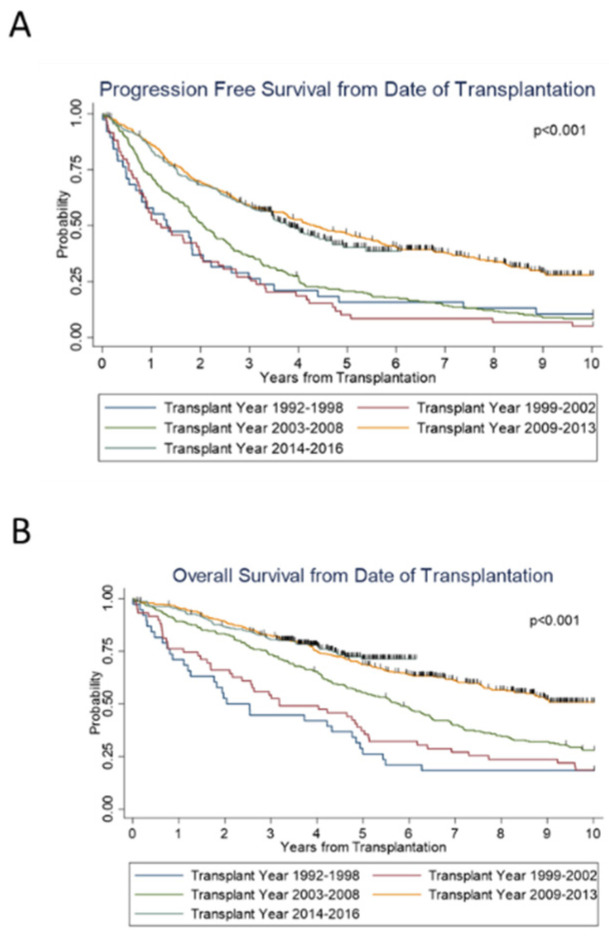
Survival outcomes from the time of transplantation by transplant years.

Across the years (1992–2016), there was a statistically significant improvement in both PFS (*p* < 0.01) and OS (*p* < 0.01). The median PFS and OS of all patients was 1.3 and 2.0 years in group 1 (1992—1998); 1.2 and 3.2 years in group 2 (1999—2002); and 2.1 and 5.8 years in group 3 (2003—2008). This response was further improved with a PFS and OS of 4.3 years and not reached (NR), respectively, in group 4 (2009–2013), and 3.8 years and NR, respectively, in group 5 (2014—2016) (Figure 1). The 5-year PFS of groups 1 through 5 was 16%, 10%, 21%, 46%, and 40%, respectively. The 5-year OS of groups 1 through 5 was 26%, 37%, 55%, 69%, and 71%, respectively.

Notably, for both groups 4 and 5, the median OS was not reached (NR). The median PFS was about 4 years for these groups. Group 3 was the most recent to reach a median OS, which was 5.8 years (95% CI 4.8–6.5 years). From the time of diagnosis, both PFS (*p* < 0.001) and OS (*p* < 0.001) significantly improved over the years.

**Figure 2:**
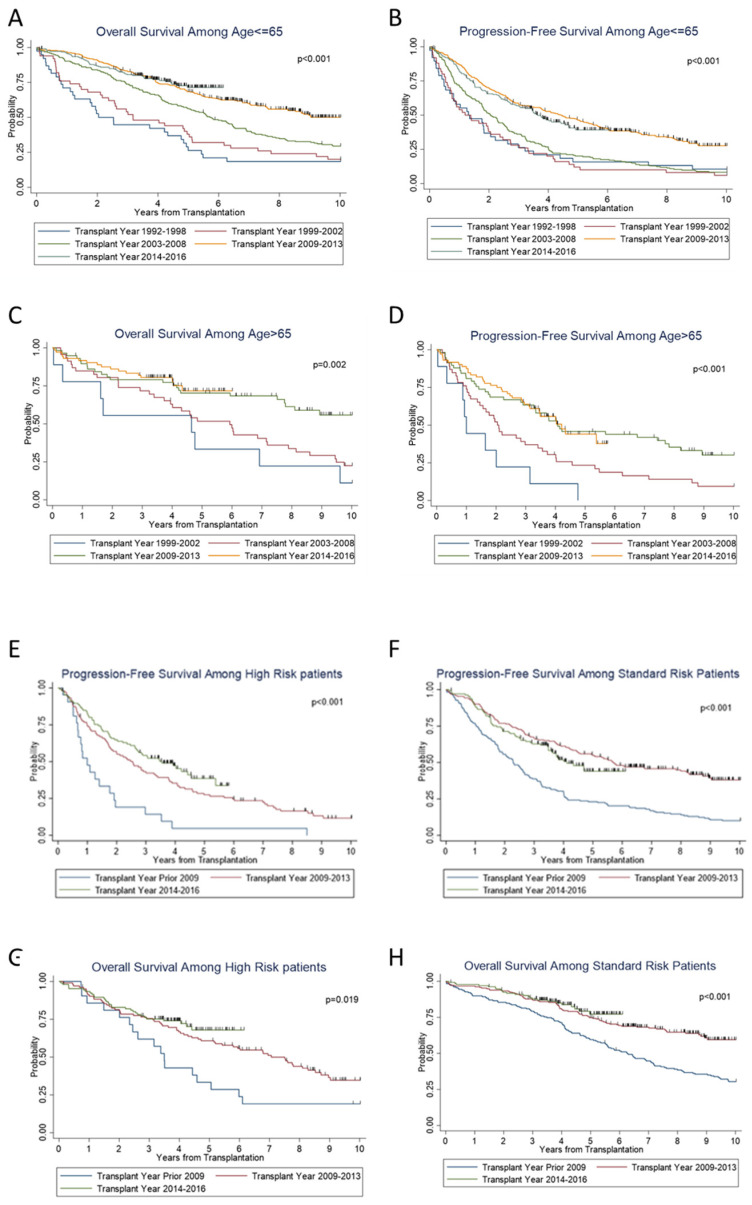
Subgroup survival outcomes and prognostic factors for survival.

**Figure 3:**
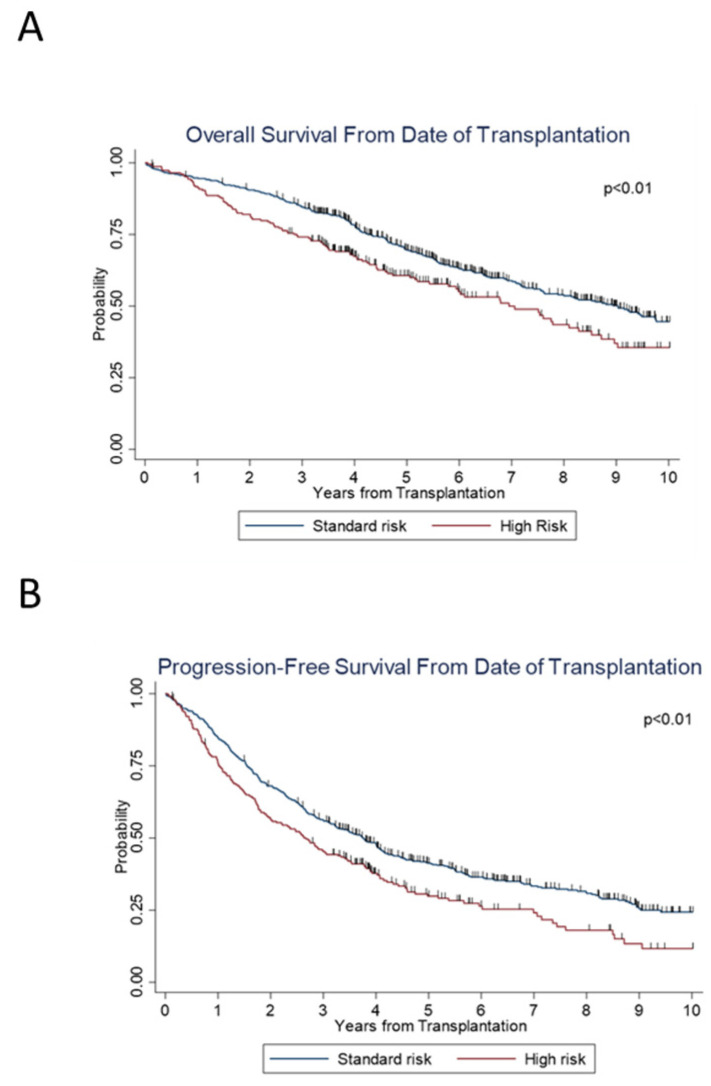
Survival outcomes between standard versus high risk patients.

We then examined the effect of age on survival. On subset analysis, across the years, significant increases in PFS (*p* < 0.001) and OS (*p* < 0.001) were seen in patients ≤ 65 years of age (Figure 2A,B), as well as in patients > 65 years old; (*p* < 0.001) and (*p* = 0.002), respectively (Figure 2C,D). We also examined the trend along the years when stratified according to standard versus high risk. For both standard- and high-risk disease, there was a significant improvement in PFS (*p* < 0.001 and *p* < 0.001) and OS (*p* = 0.02 and *p* < 0.001) (Figure 2E–H) over the years. Standard-risk patients continued to do better than high-risk patients with superior PFS (*p* < 0.01) and OS (*p* < 0.01) (Figure 3A,B).

Furthermore, the rate of response both pre- and post-transplant showed statistically significant improvement across the years (*p* < 0.01). The post-transplant response (VGPR or better) also increased from 28.9% in group 1 to 76.3% in group 5 (Table 1). Multivariable analysis showed that transplant year, pre-transplant remission status, International Staging System (ISS), and high- versus standard-risk cytogenetics have significant contributions to the risk of relapse or death (Table 2 and Table 3). Patients who received transplant between 2009–2013 and between 2014–2016 had a significantly lower risk for relapse or death, HR 0.49 (95% CI 0.38–0.62), and 0.53 (95% CI 0.40–0.69).

## 5. Discussion

Advancement in the treatment of MM has significantly improved the survival of patients, especially in the last 20 years, as novel therapies have become standard of care. Multiple factors affect outcomes, including pre-transplant response and cytogenetic risk. It has been shown that achieving complete response/very good partial response (CR/VGPR) is vital in ensuring a prolonged PFS or OS. In our study, the pre- and post-ASCT response increased from 5% to 54% and 29% to 76%, across the years. The importance of pre-transplant remission status is still controversial. In the Korean myeloma group study, CR before ASCT was associated with longer post-transplant OS (*p*=0.0015) [26]. However, in a CIBMTR-based study, for patients who failed to achieve partial response with induction treatment before ASCT, additional treatment before ASCT did not improve PFS or OS [27]. In our study, we found that pre-transplant CR or VGPR only is associated with improvement in both PFS and OS (*p* < 0.001). Post-transplant CR and/or VGPR improve PFS and OS in MM patients [28,29]. For instance, in the long-term follow-up of the GEM-PATHEMA study, post-transplant CR after ASCT remained associated with superior OS at a follow-up of 12 years (35% in CR vs. 16% in PR group; *p* < 0.001) [29]. Therefore, the achievement of CR or VGPR status post-transplant is a vital goal in ensuring a disease-free prognosis [24]. The achievement and maintenance of post-transplant CR and VGPR seen was due to the increased use of three-drug induction therapy with novel drugs, and the implementation of post-ASCT maintenance [8,15,20,22,24,30]. Better induction therapy with improved response post-ASCT are likely reasons for tandem transplant falling out of favor, which was one way to achieve deep remission in the late 1990s to early 2000s [31,32,33]. The BMT-CTN 0702 study, which randomized patients to ASCT/maintenance lenalidomide or ASCT/consolidation, VRD/lenalidomide maintenance, or tandem ASCT/lenalidomide maintenance, showed no benefit for consolidation or tandem transplant [34]. The FORTE trial evaluated the role of carfilzomib in the upfront treatment of transplant-eligible patients with NDMM [35]. The study evaluated the efficacy and safety of three different carfilzomib-based induction and consolidation approaches with or without transplantation and of maintenance treatment with carfilzomib plus lenalidomide versus lenalidomide alone in NDMM. Patients were assigned to one of the induction-consolidation groups: carfilzomib, lenalidomide, and dexamethasone (KRD), plus ASCT; KRD12; carfilzomib, cyclophosphamide, and dexamethasone (KCD), plus ASCT. The KRD12 arm did not include ASCT but instead additional cycles of KRD. The results showed that the KRD regimen plus ASCT led to deep and durable responses compared with the other two regimens. The patients in the three arms were then randomly assigned to maintenance treatment with carfilzomib plus lenalidomide or lenalidomide alone. The 3-year PFS was 75% with KRD versus 65% with lenalidomide alone (*p* = 0.023). Maintenance treatment with carfilzomib plus lenalidomide significantly improved PFS compared with lenalidomide alone.

As the population ages, there is concern about how older patients fare under the physically challenging course of treatment for their multiple myeloma disease. Less aggressive treatment regimens are often prioritized for elderly patients because of toxicity concerns. However, as the median age at diagnosis of multiple myeloma is 69 years [36], setting an age cutoff for transplant will exclude a large cohort of patients. We saw a trend of an increasing number of patients, from 0% in group 1 to 25% in group 5, who were >65 years of age at transplant. We found statistically significant improvement in PFS and OS for patient populations aged ≤65 years and >65 years (Figure 2A–D). A Swedish cohort of patients older than 70 years of age diagnosed with MM between 1973–2003 were found not to have as much improvement in 5-year relative survival ratios as their younger counterparts [37]. However, Kumar et al. demonstrated that MM patients >65 years old had a significantly longer median OS of 5 years for those diagnosed between 2006–2010, compared to 3.2 years for those between 2001–2005 [38]. Similarly, a center of international bone marrow transplant registry-based study that compared 942 patients >70 years and 10,484 younger patients showed the elderly population derives similar benefit from ASCT [39]. Although the risk of mortality increased with age, it was not due to NRM or disease progression [39]. In our study, patients >65 years old had similar 5-year OS rates compared to younger patients (≤65 years). The 5-year OS rates for older patients from groups 2–4 were 33%, 52%, and 70%, respectively, and that of the younger patients from the same groups were 38%, 56%, and 68%, respectively. Our data add to existing reports that patients should not be excluded from ASCT due to age alone.

Risk assessment in MM has become more robust and specific over time, as cytogenetic studies of patient marrow biopsies utilizing FISH have improved understanding of prognosis [40]. Combining the International Staging System (ISS), which considers serum β2-microglobulin and albumin levels, with FISH analysis of high-risk chromosomal abnormalities, was shown to significantly improve risk assessment in MM patients. This led to the creation of a revised ISS system, which has been widely accepted [41]. The International Myeloma Working Group has identified t(4;14), del(17/17p), t(14;16), t(14;20), and gain(1q) to confer poor prognosis [42]. Integration of these risk assessment factors into survival analysis can offer greater understanding of the biology of myeloma and its influence on disease course. Our analysis showed that both the standard- and high-risk patients saw improvement in PFS and OS (Figure 2E,F), although the high-risk cohort still had lower PFS and OS compared to the standard-risk cohort (Figure 3A,B).

We recognize that this is a retrospective analysis that involves only MM patients who received ASCT, which does not account for the survival of patients who were not transplant eligible or delayed ASCT after their first relapse. However, this is a large analysis showing that newer agents developed between 1992–2016, incorporated into induction therapies and maintenance, have significantly improved survival outcomes of NDMM patients receiving ASCT, with more than 80% patients surviving 3 years post-ASCT since 2009. Further studies into how demographics, access to care, and disease characteristics impact myeloma outcomes are needed to contribute to our understanding of the disease.

## 6. Conclusions

In conclusion, our study follows the outcomes of MM patients for more than 26 years. We observed that the outcomes of NDMM patients’ survival have improved dramatically since 1992, primarily due to the inclusion of novel and maintenance therapies. However, MM remains incurable. Therefore, there is hope that newer pharmacological interventions and technologies will continue to build on prior successes.

## Figures and Tables

**Table 1:** Patient Characteristics.

	ASCT Year												
	Total (n = 1001)		1992–1998 (n = 38)		1999–2002 (n = 59)		2003–2008 (n = 241)		2009–2013 (n = 376)		2014–2016 (n = 287)		*p*-Value
**Age at ASCT (median, range)**	58	18–81	54	36–65	57	39–71	57	30–76	58	18–81	60	28–75	<0.01
	*N*	%	*N*	%	*N*	%	*N*	%	*N*	%	*N*	%	<0.01
**Age ≤ 65**	816	81.5	38	100.0	50	84.7	195	80.9	318	84.6	215	74.9	
**Age > 65**	185	18.5	0	0.0	9	15.3	46	19.1	58	15.4	72	25.1	
**Gender**													0.69
Male	585	58.4	22	57.9	31	52.5	144	59.8	227	60.4	161	56.1	
Female	416	41.6	16	42.1	28	47.5	97	40.2	149	39.6	126	43.9	
**Race**													0.52
Black	141	14.1	4	10.5	7	11.9	42	17.4	49	13.0	39	13.6	
White	850	84.9	33	86.8	51	86.4	199	82.6	322	85.6	245	85.4	
Others	10	1.0	1	2.6	1	1.7	0	0.0	5	1.3	3	1.0	
**Myeloma type**													0.12
IGA	226	22.7	6	16.7	16	28.1	62	25.7	72	19.1	70	24.4	
IGG	534	53.6	25	69.4	33	57.9	128	53.1	197	52.4	151	52.6	
LC only	193	19.4	2	5.6	5	8.8	37	15.4	91	24.2	58	20.2	
Nonsecretory	24	2.4	2	5.6	3	5.3	6	2.5	10	2.7	3	1.0	
IGD	15	1.5	1	2.8	0	0.0	5	2.1	5	1.3	4	1.4	
Biclonal	2	0.2	0	0.0	0	0.0	2	0.8	0	0.0	0	0.0	
PLASMA CELL LEUKEMIA	1	0.1	0	0.0	0	0.0	1	0.4	0	0.0	0	0.0	
IGM	1	0.1	0	0.0	0	0.0	0	0.0	1	0.3	0	0.0	
IGE	1	0.1	0	0.0	0	0.0	0	0.0	0	0.0	1	0.3	
Unknown	4		2		2		0		0		0		
**ISS staging**													0.55
1	286	38.1	1	100.0	9	32.1	58	33.7	116	37.7	102	42.3	
2	244	32.5	0	0.0	9	32.1	57	33.1	99	32.1	79	32.8	
3	220	29.3	0	0.0	10	35.7	57	33.1	93	30.2	60	24.9	
Unknown	251		37		31		69		68		46		
**Cytogenetic risk**													<0.01
Standard risk	530	69.7	1	100.0	5	100.0	178	89.4	212	67.3	134	55.8	
High risk	230	30.3	0	0.0	0	0.0	21	10.6	103	32.7	106	44.2	
NA	241		37		54		42		61		47		
**Melphalan use**													<0.01
**140**	127	12.7	0	0.0	5	8.5	18	7.5	56	14.9	48	16.7	
**200**	803	80.2	2	5.3	22	37.3	220	91.3	320	85.1	239	83.3	
**Missing or No Use**	71	7.1	36	94.7	32	54.2	3	1.2	0	0.0	0	0.0	
**Pre-Transplant response**													<0.01
Others	141	14.1	12	31.6	14	23.7	33	13.7	55	14.6	27	9.4	
CR/VGPR	455	45.5	2	5.3	9	15.3	96	39.8	193	51.3	155	54.0	
PR	405	40.5	24	63.2	36	61.0	112	46.5	128	34.0	105	36.6	
**Post-Transplant response**													<0.01
Others	103	10.3	24	63.2	18	30.5	29	12.0	18	4.8	14	4.9	
CR/VGPR	708	70.7	11	28.9	18	30.5	158	65.6	302	80.3	219	76.3	
PR	190	19.0	3	7.9	23	39.0	54	22.4	56	14.9	54	18.8	
**Induction Regimens**													<0.01
Cytoxan based therapy	5	0.5	2	5.3	2	3.4	1	0.4	0	0.0	0	0.0	
MP based therapy	8	0.8	6	15.8	2	3.4	0	0.0	0	0.0	0	0.0	
VAD based therapy	128	12.8	30	78.9	54	91.5	44	18.3	0	0.0	0	0.0	
Lenalidomide based therapy	119	11.9	0	0.0	0	0.0	37	15.4	63	16.8	19	6.6	
Thalidomide and bortezomib based therapy	29	2.9	0	0.0	0	0.0	22	9.1	7	1.9	0	0.0	
Thalidomide based therapy	61	6.1	0	0.0	0	0.0	57	23.7	4	1.1	0	0.0	
bortezomib based therapy	324	32.4	0	0.0	0	0.0	66	27.4	157	41.8	101	35.2	
lenalidomide and Bortezomib based therapy	323	32.3	0	0.0	0	0.0	14	5.8	142	37.8	167	58.2	
Melphalan based regimen	1	0.1	0	0.0	0	0.0	0	0.0	1	0.3	0	0.0	
Unknown	3	0.3	0	0.0	1	1.7	0	0.0	2	0.5	0	0.0	
**Received maintenance**	472	47.2	0	0.0	0	0.0	20	8.3	254	67.6	198	69.0	<0.01
**Maintenance drugs**													<0.01
Lenalidomide	411	87.1	0	0	0	0	18	90	233	91.7	160	80.8	
Bortezomib	51	10.8	0	0	0	0	0	0	24	9.4	27	13.6	
IXAZOMIB	13	2.8	0	0	0	0	0	0	2	0.8	11	5.6	
LENOLIDIMIDE+bortezomib	6	1.3	0	0	0	0	0	0	5	2.0	1	0.5	
Lenalidomide+cyclophosphamide	6	1.3	0	0	0	0	0	0	2	0.8	4	2.0	
Pomalidomide	8	1.7	0	0	0	0	0	0	0	0.0	8	4.0	
Pomalidomide+cyclophosphamide	2	0.4	0	0	0	0	0	0	0	0.0	2	1.0	
Thalidomide	5	1.1	0	0	0	0	2	10	3	1.2	0	0.0	

**Table 2:** Cox Proportional Hazard Model on Risk of Relapse or Death.

Characteristics	HR	95% CI		*p*-Value
Univariable modelTransplant year				
Transplant year ≤ 2008	Reference			
Transplant year 2009—2013	0.48	0.40	0.57	<0.001
Transplant year 2014—2016	0.52	0.42	0.63	<0.001
Age at ASCT	1.00	0.99	1.01	0.814
Age ≤ 65	Reference			
Age > 65	0.90	0.74	1.09	0.296
Gender				
Male	Reference			
Female	0.98	0.85	1.14	0.824
Race				
Black	Reference			
White	1.01	0.82	1.25	0.923
Others	0.63	0.26	1.54	0.308
Melphalan dose (mg/m^2^)				
140	Reference			
200	0.90	0.72	1.13	0.362
Missing or No use	2.16	1.57	2.96	<0.001
Pre-transplant response				
Others	Reference			
CR/VGPR	0.44	0.36	0.55	<0.001
PR	0.75	0.61	0.93	0.007
ISS staging				
1	Reference			
2	1.16	0.94	1.43	0.156
3	1.54	1.25	1.90	<0.001
Cytogenetics risk				
Standard risk	Reference			
High risk	1.39	1.16	1.67	<0.001
Myeloma type				
IGA	Reference			
IGG	1.03	0.86	1.24	0.763
LC only	0.91	0.72	1.14	0.406
Others	1.06	0.75	1.50	0.754
Multivariable model				
Transplant years				
Transplant year ≤ 2008	Reference			
Transplant year 2009–2013	0.49	0.38	0.62	<0.001
Transplant year 2014–2016	0.53	0.40	0.69	<0.001
Pre-transplant response				
Others	Reference			
CR/VGPR	0.54	0.40	0.73	<0.001
PR	0.90	0.67	1.22	0.500
ISS staging				
1	Reference			
2	1.21	0.96	1.53	0.104
3	1.51	1.18	1.94	0.001
Cytogenetics risk				
Standard risk				
High risk	1.80	1.45	2.25	<0.001
Melphalan dose (mg/m^2^)				
140	Reference			
200	1.10	0.81	1.50	0.535
Missing or No use	14.06	1.82	108.46	0.011

**Table 3:** Cox Proportional Hazard Model on Risk of Death.

Characteristics	HR	95% CI		*p*-Value
Univariable model				
Transplant year ≤ 2008				
Transplant year 2009—2013	0.50	0.41	0.60	<0.001
Transplant year 2014—2016	0.46	0.35	0.60	<0.001
Age at ASCT	1.01	1.00	1.02	0.219
Age ≤ 65	Reference			
Age > 65	0.96	0.76	1.21	0.717
Gender				
Male	Reference			
Female	0.86	0.72	1.03	0.100
Race				
Black	Reference			
White	1.07	0.83	1.39	0.589
Others	0.70	0.22	2.24	0.553
Melphalan dose (mg/m^2^)				
140	Reference			
200	0.74	0.57	0.96	0.026
Missing or No use	2.18	1.55	3.06	<0.001
Pre-transplant response				
Others	Reference			
CR/VGPR	0.45	0.35	0.58	<0.001
PR	0.83	0.65	1.05	0.120
ISS staging				
1	Reference			
2	1.37	1.05	1.79	0.020
3	2.03	1.57	2.63	<0.001
Cytogenetics risk				
Standard risk	Reference			
High risk	1.42	1.13	1.79	0.003
Myeloma type				
IGA	Reference			
IGG	0.87	0.71	1.08	0.217
LC only	0.79	0.60	1.04	0.095
Others	0.89	0.59	1.34	0.564
Multivariable model				
Transplant years				
Transplant year ≤ 2008	Reference			
Transplant year 2009–2013	0.54	0.41	0.72	<0.001
Transplant year 2014–2016	0.53	0.36	0.77	0.001
Pre-transplant remission				
Others	Reference			
CR/VGPR	0.53	0.37	0.77	0.001
PR	0.92	0.64	1.32	0.653
ISS staging				
1	Reference			
2	1.48	1.09	2.02	0.011
3	2.00	1.48	2.70	<0.001
Cytogenetics risk				
Standard risk				
High risk	1.80	1.36	2.37	<0.001

## Data Availability

The data presented in this study are available on request.

## References

[1] Kumar S.K., Rajkumar S.V., Dispenzieri A., Lacy M.Q., Hayman S.R., Buadi F.K., Zeldenrust S.R., Dingli D., Russell S.J., Lust J.A. (2008). Improved survival in multiple myeloma and the impact of novel therapies. Blood.

[2] Turesson I., Velez R., Kristinsson S.Y., Landgren O. (2010). Patterns of Improved Survival in Patients with Multiple Myeloma in the Twenty-First Century: A Population-Based Study. J. Clin. Oncol..

[3] Anderson K.C. (2016). Progress and Paradigms in Multiple Myeloma. Clin. Cancer Res..

[4] Attal M., Harousseau J.-L., Stoppa A.-M., Sotto J.-J., Fuzibet J.-G., Rossi J.-F., Casassus P., Maisonneuve H., Facon T., Ifrah N. (1996). A Prospective, Randomized Trial of Autologous Bone Marrow Transplantation and Chemotherapy in Multiple Myeloma. N. Engl. J. Med..

[5] Shah N., Callander N., Ganguly S., Gul Z., Hamadani M., Costa L., Sengsayadeth S., Abidi M., Hari P., Mohty M. (2015). Hematopoietic Stem Cell Transplantation for Multiple Myeloma: Guidelines from the American Society for Blood and Marrow Transplantation. Biol. Blood Marrow Transplant..

[6] Bashir Q., Shah N., Parmar S., Wei W., Rondon G., Weber D.M., Wang M., Orlowski R.Z., Thomas S.K., Shah J. (2012). Feasibility of autologous hematopoietic stem cell transplant in patients aged ≥ 70 years with multiple myeloma. Leuk. Lymphoma.

[7] Kumar S.K., Dingli D., Lacy M.Q., Dispenzieri A., Hayman S.R., Buadi F.K., Rajkumar S.V., Litzow M.R., Gertz M.A. (2008). Autologous stem cell transplantation in patients of 70 years and older with multiple myeloma: Results from a matched pair analysis. Am. J. Hematol..

[8] Paul B., Lipe B., Ocio E.M., Usmani S.Z. (2019). Induction Therapy for Newly Diagnosed Multiple Myeloma. Am. Soc. Clin. Oncol. Educ. Book.

[9] Ria R., Reale A., Solimando A.G., Mangialardi G., Moschetta M., Gelao L., Iodice G., Vacca A. (2012). Induction therapy and stem cell mobilization in patients with newly diagnosed multiple myeloma. Stem Cells Int..

[10] Latif T., Chauhan N., Khan R., Moran A., Usmani S.Z. (2012). Thalidomide and its analogues in the treatment of Multiple Myeloma. Exp. Hematol. Oncol..

[11] Cavo M., Zamagni E., Tosi P., Tacchetti P., Cellini C., Cangini D., de Vivo A., Testoni N., Nicci C., Terragna C. (2005). Superiority of thalidomide and dexamethasone over vincristine-doxorubicindexamethasone (VAD) as primary therapy in preparation for autologous transplantation for multiple myeloma. Blood.

[12] Barlogie B., Jagannath S., Desikan K.R., Mattox S., Vesole D., Siegel D., Tricot G., Munshi N., Fassas A., Singhal S. (1999). Total therapy with tandem transplants for newly diagnosed multiple myeloma. Blood.

[13] Barlogie B., Kyle R.A., Anderson K.C., Greipp P.R., Lazarus H.M., Hurd D.D., McCoy J., Moore D.F., Dakhil S.R., Lanier K.S. (2006). Standard Chemotherapy Compared with High-Dose Chemoradiotherapy for Multiple Myeloma: Final Results of Phase III US Intergroup Trial S9321. J. Clin. Oncol..

[14] Barlogie B., Jagannath S., Vesole D.H., Naucke S., Cheson B., Mattox S., Bracy D., Salmon S., Jacobson J., Crowley J. (1997). Superiority of Tandem Autologous Transplantation Over Standard Therapy for Previously Untreated Multiple Myeloma. Blood.

[15] Rajkumar S.V., Hayman S.R., Lacy M.Q., Dispenzieri A., Geyer S.M., Kabat B., Zeldenrust S.R., Kumar S., Greipp P.R., Fonseca R. (2005). Combination therapy with lenalidomide plus dexamethasone (Rev/Dex) for newly diagnosed myeloma. Blood.

[16] Sonneveld P., Schmidt-Wolf I.G.H., van der Holt B., el Jarari L., Bertsch U., Salwender H., Zweegman S., Vellenga E., Broyl A., Blau I.W. (2012). Bortezomib Induction and Maintenance Treatment in Patients with Newly Diagnosed Multiple Myeloma: Results of the Randomized Phase III HOVON-65/ GMMG-HD4 Trial. J. Clin. Oncol..

[17] Facon T., Lee J.H., Moreau P., Niesvizky R., Dimopoulos M.A., Hajek R., Osman M., Aggarwal S., Klippel Z., San Miguel J. (2017). Phase 3 Study (CLARION) of Carfilzomib, Melphalan, Prednisone (KMP) v Bortezomib, Melphalan, Prednisone (VMP) in Newly Diagnosed Multiple Myeloma (NDMM). Clin. Lymphoma Myeloma Leuk..

[18] Tosi P., Zamagni E., Tacchetti P., Ceccolini M., Perrone G., Brioli A., Pallotti M.C., Pantani L., Petrucci A., Baccarani M. (2010). Thalidomide-dexamethasone as induction therapy before autologous stem cell transplantation in patients with newly diagnosed multiple myeloma and renal insufficiency. Biol. Blood Marrow Transplant..

[19] Cavo M., Tacchetti P., Patriarca F., Petrucci M.T., Pantani L., Galli M., Di Raimondo F., Crippa C., Zamagni E., Palumbo A. (2010). Bortezomib with thalidomide plus dexamethasone compared with thalidomide plus dexamethasone as induction therapy before, and consolidation therapy after, double autologous stem-cell transplantation in newly diagnosed multiple myeloma: A randomised phase 3 study. Lancet.

[20] Rosiñol L., Oriol A., Teruel A.I., Hernández D., López-Jiménez J., de la Rubia J., Granell M., Besalduch J., Palomera L., González Y. (2012). Superiority of bortezomib, thalidomide, and dexamethasone (VTD) as induction pretransplantation therapy in multiple myeloma: A randomized phase 3 PETHEMA/GEM study. Blood.

[21] Durie B.G.M., Hoering A., Abidi M.H., Rajkumar S.V., Epstein J., Kahanic S.P., Thakuri M., Reu F., Reynolds C.M., Sexton R. (2017). Bortezomib with lenalidomide and dexamethasone versus lenalidomide and dexamethasone alone in patients with newly diagnosed myeloma without intent for immediate autologous stem-cell transplant (SWOG S0777): A randomised, open-label, phase 3 trial. Lancet.

[22] Attal M., Lauwers-Cances V., Hulin C., Leleu X., Caillot D., Escoffre M., Arnulf B., Macro M., Belhadj K., Garderet L. (2017). Lenalidomide, Bortezomib, and Dexamethasone with Transplantation for Myeloma. N. Engl. J. Med..

[23] Dhakal B., Szabo A., Chhabra S., Hamadani M., D’Souza A., Usmani S.Z., Sieracki R., Gyawali B., Jackson J.L., Asimakopoulos F. (2018). Autologous Transplantation for Newly Diagnosed Multiple Myeloma in the Era of Novel Agent Induction: A Systematic Review and Meta-analysis. JAMA Oncol..

[24] McCarthy P.L., Owzar K., Hofmeister C.C., Hurd D.D., Hassoun H., Richardson P.G., Giralt S., Stadtmauer E.A., Weisdorf D.J., Vij R. (2012). Lenalidomide after stem-cell transplantation for multiple myeloma. N. Engl. J. Med..

[25] Voorhees P.M., Kaufman J.L., Laubach J., Sborov D.W., Reeves B., Rodriguez C., Chari A., Silbermann R., Costa L.J., Anderson L.D. (2020). Daratumumab, lenalidomide, bortezomib, and dexamethasone for transplant-eligible newly diagnosed multiple myeloma: The GRIFFIN trial. Blood.

[26] Kim J.S., Kim K., Cheong J.-W., Min Y.H., Suh C., Kim H., Jo D.Y., Ryoo H.M., Yoon S.S., Lee J.H. (2009). Complete Remission Status before Autologous Stem Cell Transplantation Is an Important Prognostic Factor in Patients with Multiple Myeloma Undergoing Upfront Single Autologous Transplantation. Biol. Blood Marrow Transplant..

[27] Vij R., Kumar S., Zhang M.-J., Zhong X., Huang J., Dispenzieri A., Abidi M.H., Bird J.M., Freytes C.O., Gale R.P. (2015). Impact of Pretransplant Therapy and Depth of Disease Response before Autologous Transplantation for Multiple Myeloma. Biol. Blood Marrow Transplant..

[28] Harousseau J.-L., Avet-Loiseau H., Attal M., Charbonnel C., Garban F., Hulin C., Michallet M., Facon T., Garderet L., Marit G. (2009). Achievement of at Least Very Good Partial Response Is a Simple and Robust Prognostic Factor in Patients with Multiple Myeloma Treated with High-Dose Therapy: Long-Term Analysis of the IFM 99-02 and 99-04 Trials. J. Clin. Oncol..

[29] Martinez-Lopez J., Blade J., Mateos M.-V., Grande C., Alegre A., García-Laraña J., Sureda A., de la Rubia J., Conde E., Martinez R. (2011). Long-term prognostic significance of response in multiple myeloma after stem cell transplantation. Blood.

[30] Attal M., Lauwers-Cances V., Marit G., Caillot D., Moreau P., Facon T., Stoppa A.M., Hulin C., Benboubker L., Garderet L. (2012). Lenalidomide maintenance after stem-cell transplantation for multiple myeloma. N. Engl. J. Med..

[31] Attal M., Harousseau J.-L., Facon T., Guilhot F., Doyen C., Fuzibet J.-G., Monconduit M., Hulin C., Caillot D., Bouabdallah R. (2003). Single versus Double Autologous Stem-Cell Transplantation for Multiple Myeloma. N. Engl. J. Med..

[32] Cavo M., Tosi P., Zamagni E., Cellini C., Tacchetti P., Patriarca F., Di Raimondo F., Volpe E., Ronconi S., Cangini D. (2007). Prospective, randomized study of single compared with double autologous stem-cell transplantation for multiple myeloma: Bologna 96 clinical study. J. Clin. Oncol..

[33] Sonneveld P., van der Holt B., Segeren C.M., Vellenga E., Croockewit A.J., Verhoe G.E., Cornelissen J.J., Schaafsma M.R., van Oers M.H., Wijermans P.W. (2007). Intermediate-dose melphalan compared with myeloablative treatment in multiple myeloma: Long-term follow-up of the Dutch Cooperative Group HOVON 24 trial. Haematologica.

[34] Stadtmauer E.A., Pasquini M.C., Blackwell B., Hari P., Bashey A., Devine S., Efebera Y., Ganguly S., Gasparetto C., Geller N. (2019). Autologous Transplantation, Consolidation, and Maintenance Therapy in Multiple Myeloma: Results of the BMT CTN 0702 Trial. J. Clin. Oncol..

[35] Gay F., Musto P., Rota-Scalabrini D., Bertamini L., Belotti A., Galli M., Offidani M., Zamagni E., Ledda A., Grasso M. (2021). Carfilzomib with cyclophosphamide and dexamethasone or lenalidomide and dexamethasone plus autologous transplantation or carfilzomib plus lenalidomide and dexamethasone, followed by maintenance with carfilzomib plus lenalidomide or lenalidomide alone for patients with newly diagnosed multiple myeloma (FORTE): A randomised, open-label, phase 2 trial. Lancet Oncol..

[36] Ludwig H., Bolejack V., Crowley J., Bladé J., Miguel J.S., Kyle R.A., Rajkumar S.V., Shimizu K., Turesson I., Westin J. (2010). Survival and Years of Life Lost in Different Age Cohorts of Patients with Multiple Myeloma. J. Clin. Oncol..

[37] Kristinsson S.Y., Landgren O., Dickman P.W., Derolf Å.R., Björkholm M. (2007). Patterns of Survival in Multiple Myeloma: A Population-Based Study of Patients Diagnosed in Sweden from 1973 to 2003. J. Clin. Oncol..

[38] Kumar S.K., Dispenzieri A., Lacy M.Q., Gertz M.A., Buadi F.K., Pandey S., Kapoor P., Dingli D., Hayman S.R., Leung N. (2014). Continued improvement in survival in multiple myeloma: Changes in early mortality and outcomes in older patients. Leukemia.

[39] Sharma M., Zhang M.-J., Zhong X., Abidi M.H., Akpek G., Bacher U., Callander N.S., Dispenzieri A., Freytes C.O., Fung H.C. (2014). Older patients with myeloma derive similar benefit from autologous transplantation. Biol. Blood Marrow Transplant..

[40] Neben K., Jauch A., Bertsch U., Heiss C., Hielscher T., Seckinger A., Mors T., Müller N.Z., Hillengass J., Raab M.S. (2010). Combining information regarding chromosomal aberrations t(4;14) and del(17p13) with the International Staging System classification allows stratification of myeloma patients undergoing autologous stem cell transplantation. Haematologica.

[41] Avet-Loiseau H., Durie B.G., Cavo M., Attal M., Gutierrez N., Haessler J., Goldschmidt H., Hajek R., Lee J.H., Sezer O. (2013). Combining fluorescent in situ hybridization data with ISS staging improves risk assessment in myeloma: An International Myeloma Working Group collaborative project. Leukemia.

[42] Sonneveld P., Avet-Loiseau H., Lonial S., Usmani S., Siegel D., Anderson K.C., Chng W.-J., Moreau P., Attal M., Kyle R.A. (2016). Treatment of multiple myeloma with high-risk cytogenetics: A consensus of the International Myeloma Working Group. Blood.

